# *Rhodococcus erythropolis* MTHt3 biotransforms ergopeptines to lysergic acid

**DOI:** 10.1186/s12866-015-0407-7

**Published:** 2015-03-28

**Authors:** Michaela Thamhesl, Elisabeth Apfelthaler, Heidi Elisabeth Schwartz-Zimmermann, Elisavet Kunz-Vekiru, Rudolf Krska, Wolfgang Kneifel, Gerd Schatzmayr, Wulf-Dieter Moll

**Affiliations:** BIOMIN Research Center, Technopark 1, 3430 Tulln, Austria; Department for Agrobiotechnology (IFA-Tulln), Christian Doppler Laboratory for Mycotoxin Research, Center for Analytical Chemistry, University of Natural Resources and Life Sciences (BOKU) Vienna, Konrad Lorenz Straße 20, 3430 Tulln, Austria; Christian Doppler Laboratory for Innovative Bran Biorefinery, University of Natural Resources and Life Sciences, Muthgasse 18, 1190 Vienna, Austria

## Abstract

**Background:**

Ergopeptines are a predominant class of ergot alkaloids produced by tall fescue grass endophyte *Neotyphodium coenophialum* or cereal pathogen *Claviceps purpurea*. The vasoconstrictive activity of ergopeptines makes them toxic for mammals, and they can be a problem in animal husbandry.

**Results:**

We isolated an ergopeptine degrading bacterial strain, MTHt3, and classified it, based on its 16S rDNA sequence, as a strain of *Rhodococcus erythropolis* (*Nocardiaceae, Actinobacteria*). For strain isolation, mixed microbial cultures were obtained from artificially ergot alkaloid-enriched soil, and provided with the ergopeptine ergotamine in mineral medium for enrichment. Individual colonies derived from such mixed cultures were screened for ergotamine degradation by high performance liquid chromatography and fluorescence detection. *R. erythropolis* MTHt3 converted ergotamine to ergine (lysergic acid amide) and further to lysergic acid, which accumulated as an end product. No other tested *R. erythropolis* strain degraded ergotamine. *R. erythropolis* MTHt3 degraded all ergopeptines found in an ergot extract, namely ergotamine, ergovaline, ergocristine, ergocryptine, ergocornine, and ergosine, but the simpler lysergic acid derivatives agroclavine, chanoclavine, and ergometrine were not degraded. Temperature and pH dependence of ergotamine and ergine bioconversion activity was different for the two reactions.

**Conclusions:**

Degradation of ergopeptines to ergine is a previously unknown microbial reaction. The reaction end product, lysergic acid, has no or much lower vasoconstrictive activity than ergopeptines. If the genes encoding enzymes for ergopeptine catabolism can be cloned and expressed in recombinant hosts, application of ergopeptine and ergine degrading enzymes for reduction of toxicity of ergot alkaloid-contaminated animal feed may be feasible.

## Background

Ergot alkaloids occur widely in nature because some ergot alkaloid-producing fungi form intricate associations with plants. Such associations range from mutualistic endosymbiotic life of *Neotyphodium* or *Epichloë* species as endophytes in certain grasses [1], and *Periglandula* species in morning glory [2], to parasitic association of *Claviceps* species with rye, sorghum and other cereal plants [3]. Ergot alkaloids in plants naturally discourage mammals from feeding, which is one of the proposed advantages for plants to harbour endophytic fungi. The underlying toxicity of ergot alkaloids can be a problem in animal husbandry, and a detoxification technology to increase nutritive value and safety of animal feed would be beneficial.

**Figure 1:**
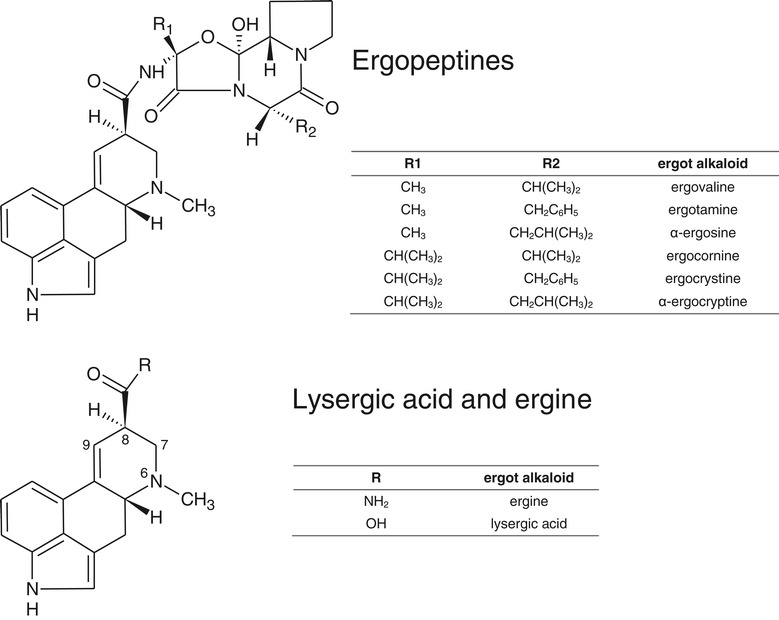
**Structures of ergopeptines, lysergic acid and ergine.**

Ergopeptines typically represent a major proportion of total ergot alkaloids both in sclerotia, which are hard, pigmented mycelium structures formed by *Claviceps purpurea* as wintering bodies on cereal ears, and in endophyte infected grass. In ergopeptines, cyclic tripeptides are linked to D-lysergic acid by an amide bond (Figure 1). There is tryptophan at position two of the tripeptide in ergotamine (one of the predominant ergopeptines in sclerotia of *C. purpurea*), whereas there is valine at this position in ergovaline (the predominant ergopeptine in endophyte-infected grass) [4]. Lysergic acid derivatives, including ergopeptines, can undergo epimerisation at the chiral carbon atom C-8. The left-hand rotation isomers (C-8(R) configuration) are pharmacologically active, and are named with the suffix –ine (e.g., ergotamine) and the right-hand rotation isomers (C-8(S) configuration) have lower or no pharmacological activity, and are named with the suffix –inine (e.g., ergotaminine) [5]. Pharmacological activity is based on structural similarity to neurotransmitters serotonin, dopamine, epinephrine or norepinephrine [6], and ergot alkaloids have found a number of medical uses including treatment of migraine [7], Parkinson’s disease [8], prolactin-related disorders [9], or in obstetrics [10,11]. One important effect of ergot alkaloids in mammals is vasoconstriction: Typical symptoms of ergot alkaloid poisoning of humans in previous centuries included skin discoloration and gangrene of hands or feet, caused by constriction of blood vessels, and called St. Anthony’s Fire. Fescue toxicosis, a kind of poisoning that animals may display after consumption of ergot alkaloids by grazing on pastures of tall fescue (*Festuca arundinacea*) infected with *Neotyphodium coenophialum*, typically includes symptoms that are related to restricted blood flow in the peripheral vascular system. Such symptoms are changes in body temperature and respiration rate, fat necrosis, dry gangrene or necrosis of tail tips, ear tips or hooves, and may even include loss of hooves [12-14]. Ergopeptines were found to elicit strong vasoconstriction, whereas lysergic acid caused much less or no contractile response [15-19]. An agronomically important symptom of ergot alkaloid poisoning, reduced feed intake and reduced weight gain, has also been related to vasoconstriction [17,19,20].

Ergot alkaloid poisoning of humans is mostly considered a problem of the past, because sclerotia of *C. purpurea* in rye or other grains can be removed by grain cleaning in mills. In animal nutrition, ergot alkaloid poisoning mostly concerns ruminants grazing on endophyte infected pastures of tall fescue. The economic loss caused by ergot alkaloids in animal husbandry in the United States was estimated in 1993 to be $ 600 million per year [21], and is likely to have increased to over $ 1 billion per year [13,22]. It has been attempted to increase the tolerance of animals to ergot alkaloids on tall fescue pastures by providing adsorbents as feed additives [23-26]. As an alternative, biological detoxification through microbial or enzymatic degradation has been investigated for other mycotoxins [27-29]. Ergot alkaloids were also found to be susceptible to microbial degradation, during rumen fermentation [30] or in earthworm intestine [31]. Bioconversion of ergine to lysergic acid by a *Rhodococcus equi* strain, in the meantime re-classified as *R. erythropolis* [32], has been reported [33], but microbial strains that can transform or metabolise ergopeptines have never been described.

The aim of our work was to explore microbial and enzymatic detoxification of ergot alkaloids with the long term goal of developing a feed additive to ameliorate toxicity of ergot alkaloid-contaminated feed. In our screening for ergot alkaloid-degrading microbial strains, we used ergotamine and ergine as substrates, because both substances were commercially available and both substances may be responsible for ergot alkaloid-toxicosis because of their vasoconstrictive activity. We isolated a new bacterial strain, *Rhodococcus erythropolis* MTHt3, which converted ergopeptines to ergine and further to lysergic acid. Characterisation of the strain’s ergopeptine conversion activity suggested that the reactions are likely catalysed by two separate enzymes, which may, if they are identified and produced in recombinant hosts, be suitable for an application to reduce toxicity of ergot alkaloid-contaminated animal feed.

## Results

### Isolation and taxonomic assignment of strain MTHt3

We attempted to isolate ergot alkaloid-degrading microbial strains from samples of soil, rumen fluid, and mites and weevils which infested our stock of sclerotia. Degradation of the ergot alkaloids used as screening substrate, ergotamine and ergine, was repeatedly observed when sample resuspensions in various growth media or buffers were incubated with substrate under aerobic or anaerobic conditions. Biological activity could be distinguished from substrate adsorption by using autoclaved samples as negative controls and by recording time curves. However, none of the strains isolated from such habitats, directly or after enrichment, showed ergotamine degradation activity, and only one of the strains degraded ergine. We also screened 98 bacterial strains and 96 yeast strains from a local, proprietary strain collection for ergotamine or ergine bioconversion activity. Five bacterial and five yeast strains with ergine bioconversion activity, but no strains with ergotamine bioconversion activity, were found. The product of ergine bioconversion was always lysergic acid.

**Figure 2:**
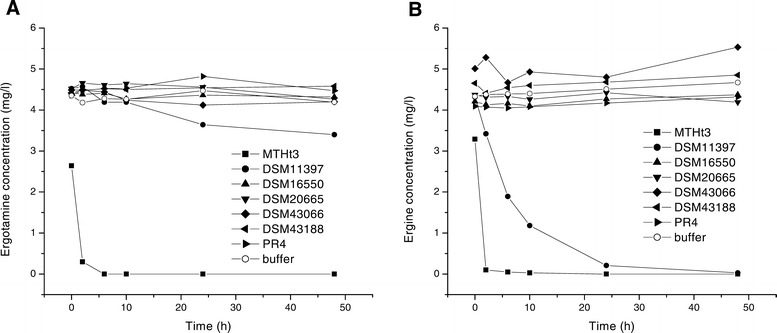
**Concentrations of ergotamine (A) and ergine (B) after incubation with** ***Rhodococcus erythropolis*** **strains or buffer.** Reactions contained biomass of *R. erythropolis* strains at OD_600nm_ = 2.0, which was equivalent to 31% (MTHt3, DSM 20665, DSM 43188) to 80% (DSM 11397) of the maximum OD_600nm_ reached in TSB, in sodium phosphate buffer pH 7.0 with 5 mg/l ergotamine or ergine, and were incubated at 25°C with shaking. Strains are referenced in Table 1.

A sample of soil, in which ground sclerotia had been buried three months earlier, was resuspended in M2 buffer, which provides no source of nitrogen, and used for degradation experiments. This soil suspension showed degradation of repeatedly added doses of ergotamine. The first two of four doses of ergotamine, which were added in three days intervals, were only partially degraded. After addition of the third or fourth dose of ergotamine, in parallel samples, ergotamine concentration was reduced to zero within the three days interval. However, cell densities in these mixed culture biotransformation reactions remained low, and no change in optical absorption was observed. The cultures were plated on M2 agar with ergotamine. Colonies and microbial film grown on such plates were washed off and resuspended in M2 buffer with ergotamine, and ergotamine conversion was measured. Reduction of ergotamine concentration in these resuspensions suggested that active strains were able to grow on M2-ergotamine-agar. Two of 158 individually tested colonies showed ergotamine and also ergine degradation. The isolates in the two colonies had identical 16S rDNA sequence and were considered sister colonies of the same strain, coded MTHt3. The 16S rDNA sequence was identical with annotated 16S rDNA sequence (1520 bp) in the whole genome sequence of *Rhodococcus erythropolis* PR4 (GenBank NC_012490), an alkane degrading strain [34], and of *R. erythropolis* CCM 2595 (GenBank NC_022115), a phenol degrading strain [35]. There was a mismatch of one base compared with 16S rDNA of *R. erythropolis* type strain DSM 43066^T^ (GenBank X79289): Position 25, according to numbering of PR4 16S rDNA as annotated, is G in DSM 43066^T^, but C in MTHt3, PR4 and CCM 2595. Strain DSM 44595, originally deposited as type strain of *Nocardia coeliaca* but re-classified as *R. erythropolis*, had its 16S rDNA sequenced as part of an effort to make missing sequences of named species available [36], and it is also identical to MTHt3. Compared with the 1520 bp 16S rDNA sequence of MTHt3, type strains of other species had, in the available length of their 16S sequences, 9 mismatches (*R. qingshengii* djl-6^T^, GenBank DQ090961, and *R. jialingiae* djl-6-2^T^, GenBank DQ185597), 12 mismatches (*R. baikonurensis* GTC 1041 T, GenBank AB071951), 23 mismatches (*R. globerulus* DSM 4954, GenBank X80619), or more mismatches. None of the other *R. erythropolis* strains (Table 1) we tested with high biomass concentration and long incubation time converted ergotamine. One strain (DSM 11397) transformed ergine to lysergic acid, but the reaction was slower than with strain MTHt3 (Figure 2).

**Table 1:** **List of** ***Rhodococcus*** **strains**

**Strain**	**Properties**	**Reference**
*R. erythropolis* MTHt3 (DSM 25948)	degrades ergot alkaloids	present work
*R. erythropolis* DSM 11397	nitrile hydratase (NHase) producer	Layh et al. [49]
*R. erythropolis* DSM 20665	degrades picolilic acid	Koch et al. [77]
*R. erythropolis* DSM 43066^T^	type strain	Goodfellow and Alderson [78]
*R. erythropolis* DSM 43188	fixes nitrogen	Metcalfe and Brown [79]
*R. erythropolis* PR4	degrades alkanes	Komukai-Nakamura et al. [80]
*Rhodococcus* sp. DSM 16550 (previously classified as *R. erythropolis*)	degrades 1-haloalkanes	Kulakova et al. [81]

### Characterisation of ergot alkaloid-biodegradation by MTHt3

**Figure 3:**
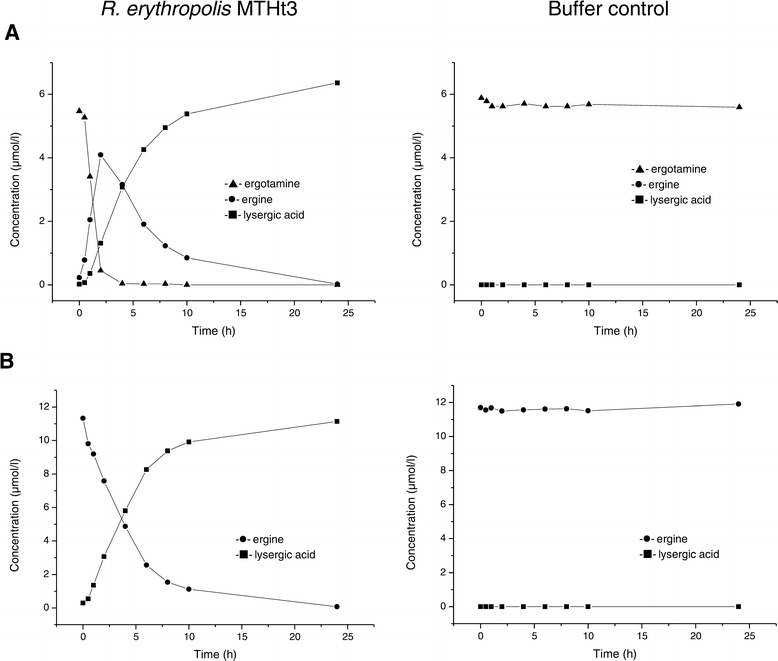
**Concentration of ergot alkaloids after incubation of ergotamine (A) or ergine (B) with** ***R. erythropolis*** **MTHt3, or buffer alone.** Biomass concentration: 2% of maximum cell density reached in TSB; buffer: Teorell-Stenhagen buffer pH 7.0; ergot alkaloid starting concentration: 5 mg/l (=8.6 μM ergotamine or 18.7 μM ergine). Plotted data are from single biotransformation reactions.

**Figure 4:**
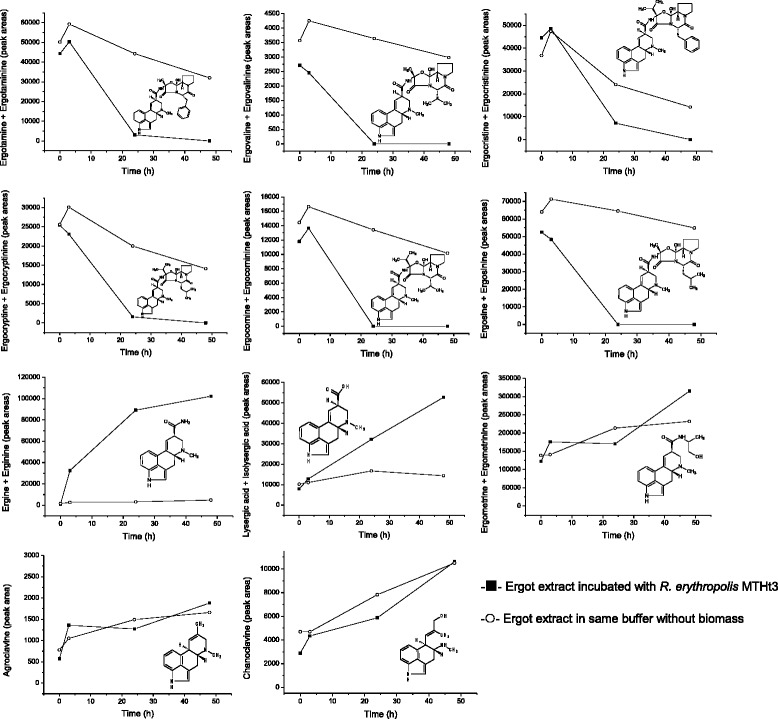
**Concentration of ergot alkaloids in** ***Claviceps purpurea*** **ergot-extract incubated with** ***R. erythropolis*** **MTHt3.** Extract was incubated with *R. erythropolis* MTHt3 (■) at 1.6% of maximum cell density reached in TSB, or in the same sodium phosphate buffer without biomass (○) (data from single flasks plotted; peak areas determined by LC/ESI-MS/MS; not calibrated).

**Figure 5:**
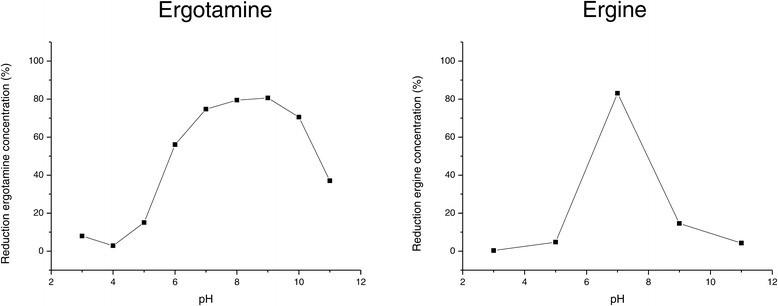
**Dependance of ergotamine and ergine biotransformation on pH.** *R. erythropolis* MTHt3 at 2% of maximum cell density reached in TSB was incubated with 5 mg/ml ergotamine or ergine in Teorell-Stenhagen buffer at various pH at 25°C. Samples from ergotamine biotransformation reactions were taken after 2 h, and samples from ergine biotransformation reactions were taken after 10 h.

**Figure 6:**
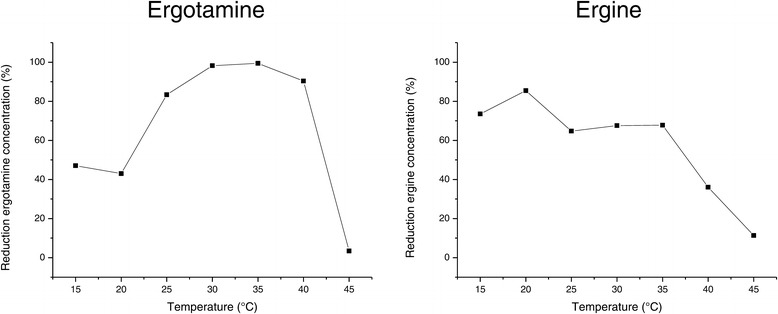
**Dependance of ergotamine and ergine biotransformation on temperature.** *R. erythropolis* MTHt3 at 2% of maximum cell density reached in TSB was incubated with 5 mg/ml ergotamine or ergine in sodium phosphate buffer pH 7.0 at various temperatures. Samples from ergotamine biotransformation reactions were taken after 2 h, and samples from ergine biotransformation reactions were taken after 10 h.

To record a time course of ergotamine and ergine bioconversion, *R. erythropolis* MTHt3-biomass was resuspended at lower concentration, 2% of the maximum OD_600_ reached in tryptone soya broth (TSB). Ergotamine was degraded quickly, ergine appeared as an intermediate produced by ergotamine degradation, and ergine was converted, more slowly, to lysergic acid (Figure 3). The data shown in Figure 3 are from single biotransformation reactions, but previous biotransformation reactions with different biomass concentrations and sampling times showed similar results. The final lysergic acid concentration was about equimolar to the ergotamine or ergine starting concentration. In order to find out which ergot alkaloids other than the ones used as screening substrates for strain isolation are degraded by *R. erythropolis* MTHt3, an ergot alkaloid extract from *Claviceps purpurea* sclerotia was prepared, and MTHt3 was incubated with this extract (Figure 4). Absolute quantification of ergot alkaloid concentrations was difficult because of an apparent increase of concentration over the course of incubation due to buffer evaporation in sample and negative control. Also, concentrations of ergot alkaloids and their corresponding C-8 epimeric –inine forms [5] had to be summed up as peak areas because the quantities of analytical standards we had available for each substance were not enough for method calibration and accurate quantification. Of the ergot alkaloids that were detectable with a multi-mycotoxin analysis method [37], *R. erythropolis* MTHt3 catalysed bioconversion of all ergopeptines, but not of the lysergic acid derivatives ergometrine (also called ergonovine or ergobasine), agroclavine and chanoclavine. As in the experiment with pure ergotamine (Figure 3), ergopeptine degradation was faster than ergine degradation. No major differences between the transformation rates of the various ergopeptines were apparent. Presumably due to the low concentration of biomass used for reaction with the ergot-extract, ergine concentration increased rather than decreased over the observed incubation period, similar to the concentration of lysergic acid. Correlations of ergotamine and ergine bioconversion activities with pH and with temperature are shown in Figures 5 and 6. Ergotamine degradation activity was high in the range of pH 7.0 to 10.0 and 25°C to 42°C. Ergine degradation activity was high at pH 7.0, and below 35°C. Both reactions proceeded only slowly at pH 5.0 or below.

## Discussion

Availability of an ergot alkaloid-degrading bacterial strain might be the starting point for an application in animal nutrition to ameliorate fescue toxicosis of grazing ruminants or ergot alkaloid poisoning of other animals. Fescue toxicosis is a considerable concern in animal husbandry and since several ways of suppressing toxicosis [14], including binding agents for use as feed additives [24], have been investigated, such an application would likely be met with demand from some farmers. Considering a feed additive application for *Rhodococcus erythropolis* MTHt3, or its enzymes, several questions emerge: Would the strain be considered a biological hazard or safe? Is ergot alkaloid-degradation a metabolic activity that is shown by many microbial strains, or is the activity unique for *R. erythropolis* MTHt3? Which of the different ergot alkaloids produced by *Claviceps purpurea*, *Neotyphodium coenophialum*, or other fungi, are degraded by the strain and which are persistent? What are the end products of degradation, and will degradation also reduce toxicity? Could an application of the strain as live microbial feed additive be considered, and would activity in animal gastrointestinal tract be high enough to allow degradation of ergot alkaloids before they are absorbed? Would it make sense to attempt cloning of the genes responsible for degradation, and consider production of enzymes for use as feed additive in recombinant hosts?

We repeatedly observed reduction of added ergotamine concentration in some but not all mixed cultures derived from environmental samples. Although we could not isolate more ergopeptine degrading strains from such cultures, microbial ergopeptine degradation activity is likely not unique to *R. erythropolis* MTHt3, but may be more widespread in the environment. *R. erythropolis* MTHt3 or other ergotamine-biotransforming strains may have been able to propagate in our enrichment cultures, because subsequent but not initial doses of ergotamine were consumed in the three day incubation period, but if any growth has occurred it was too low for changes in optical density of the culture to record. Possible effects of acetonitrile, which was used as solvent for ergotamine, were not investigated. A database search with the 16S rDNA sequence of strain MTHt3 showed that the strain can be classified with certainty as belonging to the genus *Rhodococcus*, and probably to the species *R. erythropolis. Rhodococci* are aerobic, gram-positive, non-motile nocardioform actinomycetes [38]. Members of the genus *Rhodococcus* are widespread in the environment and most common within the microbiota of soil [39]. There are various applications of members of the genus *Rhodococcus* in industrial and environmental biotechnology, mostly for bioremediation and biodegradation [39-43], but also for bioflocculant and acrylamide production [43]. Some isolates of *Rhodococcus* species were also described as capable of degrading mycotoxins aflatoxin B_1_ [44,45], T2 toxin [46] and zearalenone [47,48], and hydrolysis of ergine to lysergic acid has been reported for *R. equi* A4 [33] (re-classified *R. erythropolis* [32]). In the present work, two more *Rhodococcus* isolates catalysed this ergine to lysergic acid hydrolysis reaction: Our new isolate *R. erythropolis* MTHt3, and one of our reference strains, *R. erythropolis* DSM 11397, which has been described as nitrile hydratase (NHase) producer [49]. However, degradation or biotransformation of ergopeptines by an isolated microbial strain has, to our knowledge, never been reported before. Our comparison with reference strains showed, that ergopeptine degradation was specific for isolate MTHt3, and not a general physiological activity of *R. erythropolis*.

*R. erythropolis* is not known to be a pathogen of animals, plants or humans. A full safety evaluation considered *R. erythropolis* strain C2 safe for use in bioremediation of oil spills [50]. The pathogenic *Rhodococcus* species, *R. equi*, a pathogen of horses, and *R. fascians*, a plant pathogen, are not closely related to MTHt3 and require virulence plasmids for pathogenicity [51,52]. *R. erythropolis* MTHt3 can be considered safe for work in the laboratory and as a possible donor of genes for heterologous expression and recombinant enzyme production, but for use as live microbial feed additive, careful safety evaluation would nevertheless have to be performed.

**Figure 7:**
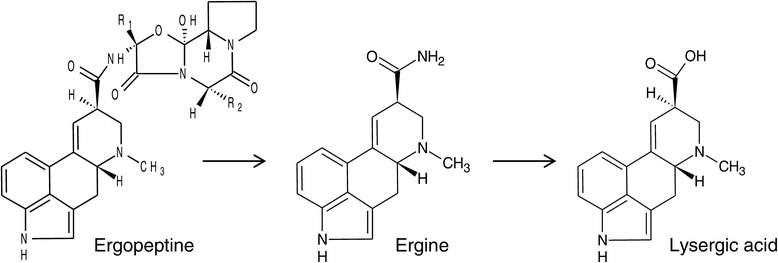
**Ergopeptine degradation pathway of** ***R. erythropolis*** **MTHt3 (R**_**1**_ **and R**_**2**_ **: see Figure** 1 **).**

Biodegradation of ergotamine seemed to be following a well-defined catabolic biotransformation pathway, with ergine as intermediate product and lysergic acid as end product (Figure 3). Incubation of *R. erythropolis* MTHt3 with an extract of *Claviceps purpurea* ergots showed that all detectable ergopeptines and ergopeptinines were biotransformed (Figure 4). The data suggest that biotransformation of all ergopeptines follows the same pathway (Figure 7), and a first enzyme in the catabolic pathway may target a molecular moiety that is shared by all ergopeptines. A second enzyme might be an amidase that converts ergine to lysergic acid. Accumulation of lysergic acid as end product of ergopeptine catabolism indicates that *R. erythropolis* MTHt3 cannot cleave the ergoline ring system. The benefit of ergopeptine catabolism for *R. erythropolis* MTHt3 may be to utilise fragments of the cyclic tripeptide moiety of ergopeptines, and the amine group of ergine, for cellular metabolism. We have started to work on isolation and identification of reaction products formed from the tripeptide moiety of ergopeptines. For the bacterium, the tripeptide moiety may be an easier target for further catabolism than the ergoline ring. It seems unlikely that after separation from the ergoline ring, fragments of the cyclic tripeptide may still have vasoconstrictive activity since no structural similarity to neurotransmitters is given, but other ergot alkaloid related or unrelated pharmacological activity cannot be ruled out.

Ergopeptines are known as the predominant and most physiologically active ergot alkaloids produced by the grass endophyte *N. coenophialum* or the cereal pathogen *C. purpurea* [4]. The biodegradation activity of *R. erythropolis* MTHt3 may therefore be able to reduce toxicity of ergot alkaloids in contaminated feed for animals. Our findings that lysergic acid accumulated as an end product of ergopeptine degradation, and that the ergot alkaloids agroclavine, chanoclavine, and ergometrine were not degraded, at least not in the complex mixture we used, pose limitations to possible animal feed detoxification applications of *R. erythropolis* MTHt3. Likely, other ergot alkaloids which are not ergopeptines will also not be degraded, and such alkaloids can also have effects on animals. Agroclavine shows antibiotic activity, interacts weakly with neurotransmitter receptors [53], and has low vasoconstrictive potential [54]. For chanoclavine, *in vitro* studies suggest a weak affinity for D-2 receptors [55], whereas *in vivo* chanoclavine has no effect [56]. Ergometrine is one of the main ergot alkaloids produced by *C. purpurea* [57]. In drunken horse grass (*Achnatherum inebrians*) infected by the endophytic fungus *N. gansuense*, high levels of ergometrine were also detected [58], but in endophyte-infected tall fescue, only minor concentrations of ergometrine were found [59]. Ergometrine stimulates uterine contractility and reduces plasma prolactin concentration for a short period after treatment [60,61]. Compared with ergopeptines, ergometrine induces a similar contractile response in a bovine lateral saphenous vein bioassay [62] and a ruminal artery assay [17], but it doesn’t have vasoconstrictive effect in a ruminal vein bioassay [17] or cytotoxic effects in cell culture [63].

In tall fescue infected with *N. coenophialum,* ergovaline is known as the predominant ergot alkaloid [64,65], but ergine can occur in similar concentrations [66], and both ergot alkaloids are converted to lysergic acid by *R. erythropolis* MTHt3. Lysergic acid is also a product of ergot alkaloid-metabolism by ruminal microorganisms [67]. Compared to ergopeptines, lysergic acid has lower toxic potential and vascular constriction was only observed with high concentrations of lysergic acid [15,16], or not at all [17,19]. *In vitro* studies indicate that ergovaline, but not lysergic acid, bioaccumulates with repetitive exposure [68], and ergopeptines are persistent in vasoconstriction, whereas lysergic acid and ergoline alkaloids including ergometrine are not [69]. The conclusion of these studies is that conversion of ergopeptines and ergine to lysergic acid should reduce toxicity.

Biodegradation with *R. erythropolis* MTHt3 may cause a transient increase of ergine concentration. Ergine was found to have vasoconstrictive activity similar to ergopeptines *in vitro* [70], but conversion of ergopeptines to ergine could have *in vivo* effects that are not obvious to predict. In summary, ergot alkaloid poisoning in animal nutrition is always caused by several alkaloids in a mixture, and composition of this mixture can vary. Biodegradation of ergopeptines with *R. erythropolis* MTHt3, or its enzymes, will likely reduce toxicity, but the degree of toxicity reduction may depend on the original composition of ergot alkaloids in a mixture.

Conversion of ergotamine to lysergic acid was fast with high biomass concentration (Figure 2), but took several hours with lower biomass concentration (Figure 3), and would probably take at least equally long *in vivo*, for instance in bovine rumen. Although release of ergot alkaloids from tall fescue in rumen fluid was reported to proceed slowly over many hours [67], and the pH (Figure 5) and temperature (Figure 6) conditions that allowed MTHt3 to biotransform ergotamine and ergine covered the range that is considered typical for bovine rumen [71,72], it doesn’t seem likely that ergopeptine degradation activity of aerobic bacterium *R. erythropolis* MTHt3 would be sufficient for detoxification in an anaerobic environment such as bovine rumen. However, an animal feed additive application based on recombinant enzymes for ergopeptine and ergine degradation might be feasible. Recombinant enzymes could potentially offer several advantages, including possibilities to use higher activity doses for faster biotransformation, to adapt to reaction conditions by enzyme engineering, to prevent accumulation of an intermediate product by tuning doses of separate enzymes to each other, and to avoid stability and safety issues associated with live strains. We are proceeding with work on identification of ergopeptine degradation genes of *R. erythropolis* MTHt3.

## Conclusions

Our isolation of *R. erythropolis* MTHt3 and characterisation of its ergot alkaloid-degradation may serve as a new starting point for future development of a technology to reduce toxicity of ergot alkaloid-contaminated animal feed, because degradation of ergopeptines by a microbial isolate has not been reported before. The strain may not be sufficiently active for use as a live microbial feed additive, but its enzymes might be suitable for detoxification, or for production of precursor molecules for pharmaceutical applications. It seems worthwhile to attempt cloning of the genes responsible for ergopeptine and ergine biotransformation.

## Methods

### Ergot alkaloids and other chemicals

Ergotamine D-tartrate was from Sigma-Aldrich (St. Louis, MO, USA). Ergine, erginine, ergotaminine, and lysergic acid were from Alfarma s.r.o. (Cernošice, Czech Republic). Glacial acetic acid (HAc), aqueous ammonia solution, and Methanol (MeOH; LC gradient grade) were from Merck (Darmstadt, Germany). HPLC-grade acetonitrile was from VWR international (West Chester, PA, USA). Ergot alkaloids were dissolved in acetonitrile for use as analytical standards or as stock solutions for degradation experiments, with the exception of ergine which was dissolved in acetonitrile/water (30/70, v/v) for preparation of stock solutions for degradation experiments. Concentrations of stock solutions for degradation experiments were 250 mg/l for ergotamine and 650 mg/l for ergine. All other chemicals were from Sigma-Aldrich (St. Louis, MO, USA).

### Isolation and cultivation of ergot alkaloid-degrading strain MTHt3

Sclerotia were obtained from an Austrian mill, where they were collected from naturally contaminated rye by an optical separation system. Sclerotia were ground (Series II® Mill, RomerLabs, Tulln, Austria), and about 200 g per spot were buried about 8 to 12 cm deep at three different spots in a field near Königsdorf, Southern Burgenland, Austria, in June. Soil samples from the spots were taken 3 months later, and 2 g portions of soil were suspended in 18 ml M2 medium spiked with 5 mg/l ergotamine as a nitrogen source. M2 medium is a modified, nitrogen-free version of Brunner mineral medium (DSMZ medium 457, German Collection of Microorganisms and Cell Cultures; http://www.dsmz.de/microorganisms/medium/pdf/DSMZ_Medium457.pdf) containing 2.44 g Na_2_HPO_4_, 1.52 g KH_2_PO_4_, 0.20 g MgSO_4_ × 7 H_2_O, 0.05 g CaCl_2_ × 2 H_2_O, 1 g glucose, and 10.0 ml trace element solution 4 (without EDTA) in 1000 ml distilled water. The soil resuspensions were shaken at 25°C and 200 rpm for 14 d, and in 3 d intervals, samples for high performance liquid chromatography and fluorescence detection (HPLC-FLD) analysis were taken, and fresh ergotamine was added to additional nominal concentration of 5 mg/l. The enrichment cultures were used to make tenfold serial dilutions. Aliquots of dilution stage 10^-2 to 10^-5 were spread on plates of M2 (containing 1.5% agar and 5 mg/l ergotamine) and incubated at 25°C until colonies appeared. Individual colonies were streaked on fresh M2-ergotamine-plates to obtain pure cultures. Single colonies from these plates were inoculated into 5 ml M2 medium with 5 mg/l ergotamine or ergine. These cultures were incubated at 25°C and 200 rpm in the dark for 7 d, and concentrations of ergotamine and ergine were determined by HPLC-FLD. Ergotamine degrading isolate MTHt3 was subsequently cultivated in TSB (Oxoid Limited, Hampshire, UK) at 25°C and 200 rpm, and cryo-conserved in this medium with 10% glycerol at −80°C. Strain MTHt3 was deposited at DSMZ with the assigned number DSM 25948.

### Strain identification

Taxonomic assignment of strain MTHt3 was initially made by PCR amplification of 16S rDNA using universal primers 27F (5′- AGA GTT TGA TCM TGG CTC AG −3′) and 1492R (5′- TAC GGY TAC CTT GTT ACG ACT T −3′) [73], and sequencing. For PCR amplification, MTHt3 was grown overnight in TSB as described, and 1 μl culture was added directly to PCR reaction buffer (10 μl 5x Phusion™ HF buffer, 200 μM of each dNTP, 0.5 μM of each primer and 1 U of Phusion™ high-fidelity DNA polymerase (Finnzymes Oy, Vantaa, Finland); final volume: 50 μl). PCR was performed in a thermocycler (Mastercycler gradient, Eppendorf, Hamburg, Germany) according to the following program: 1 cycle of 10 min at 98°C; 35 cycles of 10 s at 98°C, 20 s at 53.7°C, 45 s at 72°C; and a final extension step at 72°C for 10 min. PCR product was purified by QIAquick PCR purification kit (Qiagen) according to the manufacturer’s instructions. Sanger sequencing (primers 27F and 1492R) was performed by VBC-BIOTECH Service GmbH (Vienna, Austria). More recently, the whole genome of *Rhodococcus erythropolis* MTHt3 was sequenced to support ergopeptine biotransformation gene identification and cloning. Genomic DNA of strain MTHt3 was prepared from the biomass pellet of an overnight culture (100 ml TSB, 25°C, 200 rpm) by using Qiagen Genomic-tip 500/G kit (Hilden, Germany). Cell lysis was enhanced by addition of mutanolysin (200 U/ml) and by prolonging incubation to 4 h at 37°C. Whole-genome sequencing was performed by LGC Genomics GmbH (Berlin, Germany) using Roche 454 technology. The previously obtained 16S rDNA sequence was confirmed and extended to 1520 bp, matching the annotation of *R. erythropolis* PR4 (GenBank NC_012490) and *R. erythropolis* CCM 2595 (GenBank NC_022115) whole genome sequences. Taxonomic assignment was made by submitting 16S rDNA sequence to the ribosomal database project (https://rdp.cme.msu.edu/index.jsp [74]) for alignment with full-length 16S sequences of type strains, to EzTaxon (http://www.ezbiocloud.net/eztaxon [75]), and to BLAST (http://blast.ncbi.nlm.nih.gov/Blast.cgi).

### Biodegradation of ergotamine and ergine with other *Rhodococcus erythropolis* strains

Bacterial strains (Table 1), presently classified or at the time of use classified as *R. erythropolis*, were obtained from DSMZ (http://www.dsmz.de/). Strains were grown overnight in TSB (25°C, 200 rpm), biomass was collected by centrifugation (2 655 × g, 20°C, 5 min) and resuspended in the same volume of 50 mM sodium phosphate buffer pH 7.0. These resuspensions and the same buffer were mixed to make 10 ml aliquots with optical density (OD_600nm_) 2.0, and spiked with 5 mg/l ergotamine or ergine. Controls were the same spiked buffer without inoculation, and incubated under the same conditions (25°C, 200 rpm). Samples were taken periodically, processed as described below, and stored at −20°C until analysis by HPLC-FLD.

### Incubation of *R. erythropolis* MTHt3 with ergot extract

A suspension of MTHt3 was made by inoculating the strain in TSB and cultivating for 20 h, collecting biomass from 5 ml culture by centrifugation (2 655 × g; 10 min), and resuspending it in 5 ml 50 mM sodium phosphate buffer pH 7.0. Ergot extract was prepared by shaking 20 g ergot meal, obtained as described above, with 80 ml acetonitrile/water (1/1, v/v) for 2 h at room temperature. This crude extract was filtered (“White Ribbon Filter”, Grade 589/2: 4–12 μm; Whatman plc, Kent, UK), concentrated almost to dryness by rotary evaporation (Heidolph VV2011, Schwabach, Germany) and lyophilised (Christ, Osterode am Harz, Germany). Lyophilisate was dissolved (125 mg in 4 ml acetonitrile/water (1:1, v/v)) and filter-sterilised (Filtropur S, 0.2 μm, Sarstedt, Nümbrecht, Germany). One ml of this ergot extract was added to 0.08 ml MTHt3 suspension and 3.92 ml 50 mM sodium phosphate buffer pH 7.0, and the spiked suspension was incubated at 25°C and 200 rpm. Samples were taken and analysed by liquid chromatography/electrospray ionisation tandem mass spectrometry (LC/ESI-MS/MS) as previously described [37]. Negative control samples were taken from parallel incubation of the same, sterile ergot extract in the same buffer volume without MTHt3.

### Time course of ergotamine and ergine bioconversion

Strain MTHt3 was grown for 17 h in TSB at 25°C and 200 rpm. Biomass was harvested by centrifugation (2 655 × g, 20°C, 5 min) and resuspended in the same volume Teorell-Stenhagen universal buffer pH 7.0 (citrate, phosphate and borate; [76]). The MTHt3 suspension was diluted 1/50 with Teorell-Stenhagen universal buffer pH 7.0, spiked with 5 mg/l ergotamine or ergine, and incubated at 25°C and 200 rpm. Samples were taken periodically, processed, and analysed by HPLC-FLD as described below.

### pH and temperature dependence of ergotamine and ergine bioconversion

An overnight culture of MTHt3 was prepared as described above. Biomass was harvested by centrifugation (2 655 × g, 20°C, 5 min) and resuspended in the same volume Teorell-Stenhagen universal buffer pH 7.0 for the pH dependence experiment, or in 50 mM sodium phosphate buffer pH 7.0, for the temperature dependence experiment. Teorell-Stenhagen universal buffer prepared at pH values from 3.0 to 11.0 was spiked with ergotamine or ergine (final concentration: 5 mg/l), inoculated with 1/50 volume MTHt3 suspension, and incubated at 25°C without shaking. At the end of the incubation period, pH was measured and verified. Reference reactions were identical but without biomass. Shaking was omitted because previous results showed that shaking had no effect on ergot alkaloid-biotransformation by MTHt3, but affected buffer pH, presumably due to higher carbon dioxide exposure. For determination of correlation of activity with temperature, 50 mM sodium phosphate buffer, pH 7.0, spiked with 5 mg/l ergotamine or ergine, was set to temperatures from 15°C to 45°C by incubation in water baths, inoculated with 1/50 volume MTHt3 suspension, and further incubated without shaking. Negative controls, identical but without biomass, were incubated at the same temperatures. Samples from the pH and temperature experiments were taken at several time points and processed for HPLC-FLD analysis as described below. Time points 2 h for ergotamine biotransformation and 10 h for ergine biotransformation were chosen for the graphs in Figures 5 and 6, because biotransformations were just complete or nearly complete under the reaction conditions giving the highest activities.

### Sample analysis by high performance liquid chromatography and fluorescence detection (HPLC-FLD)

Concentrations of ergotamine and ergine, their epimeric-inine forms and lysergic acid were determined by HPLC-FLD on an HP 1100 system (Agilent, Waldbronn, Germany) equipped with a G1321A fluorescence detector. Samples from degradation experiments were centrifuged (10 621 × g, 20°C, 10 min), and 320 μl aliquots of supernatant were transferred to amber glass vials. Eighty μl acetonitrile was added, and the mixtures were vortexed and stored at −20°C until analysis. Mobile phases A (methanol:water 10/90, v/v) and B (methanol:water 80/20, v/v) contained 25 mM glacial acetic acid and were set to pH 8.0 with ammonia solution. The column thermostat of the HPLC system was kept at 20°C, the flow rate was set to 0.5 ml/min, and the injection volume to 10 μl. Elution was performed in gradient elution mode (0 min: 30% B, 2 min: 100% B, 4 min: 100% B, 4.2 min: 30% B, 7 min: 30% B for lysergic acid, ergine, and erginine; or 0 min: 30% B, 2 min: 100% B, 5.5 min: 100% B, 5.6 min: 30% B, 8.5 min: 30% B for lysergic acid, ergine, ergotamine and their epimers) on a Gemini C18 column (50 × 2 mm, 5 μm particle size; Phenomenex, Aschaffenburg, Germany). The fluorescence detector was set to excitation at 310 nm and emission at 415 nm. Concentrations of ergot alkaloids and their respective epimers were added up.

**Nucleotide sequence accession number** The 16S rDNA gene sequence of *R. erythropolis* MTHt3 was deposited at NCBI GenBank under accession number KM047507.

The study was approved by the BIOMIN R&D board.
